# Transfusion Status in Liver and Kidney Transplantation Recipients—Results from Nationwide Claims Database

**DOI:** 10.3390/jcm9113613

**Published:** 2020-11-10

**Authors:** Boyoung Park, Junghyun Yoon, Han Joon Kim, Yun Kyung Jung, Kyeong Geun Lee, Dongho Choi

**Affiliations:** 1 Department of Medicine, College of Medicine, Hanyang University, Seoul 04763, Korea; hayejine@hanyang.ac.kr; 2 Graduate School of Public Health, Hanyang University, Seoul 04763, Korea; cumyluceat@hanyang.ac.kr; 3 Department of Surgery, College of Medicine, Hanyang University, Seoul 04763, Korea; thicknyh@gmail.com (H.J.K.); jyk1986@hotmail.com (Y.K.J.); hepafel@hanyang.ac.kr (K.G.L.); 4 Hanyang ICT Fusion Medical Research Center, Seoul 04763, Korea

**Keywords:** transplantation, liver, kidney, transfusion, mortality

## Abstract

Background: This study analyzed the status and trends of transfusion and its associated factors among liver and kidney transplantation recipients. Methods: A total of 10,858 and 16,191 naïve liver or kidney transplantation recipients from 2008 to 2017 were identified through the National Health Insurance Service database. The prescription code for transfusion and the presence, number, and amount of each type of transfusion were noted. The odds ratios and 95% confidence intervals were determined to identify significant differences in transfusion and blood components by liver and kidney transplantation recipient characteristics. Results: In this study, 96.4% of liver recipients and 59.7% of kidney recipients received transfusions related to the transplantation operation, mostly platelet and fresh frozen plasma. Higher perioperative transfusion in women and declining transfusion rates from 2008 to 2017 were observed in both liver and kidney recipients. In liver recipients, the transfusion rate in those who received organs from deceased donors was much higher than that in those who received organs from living donors; however, the mortality rate according to transfusion was higher only in recipients of deceased donor organs. In kidney recipients, a higher mortality rate was observed in those receiving transfusion than that in patients without transfusion. Conclusions: In Korea, the transfusion rates in liver and kidney recipients were relatively higher than those in other countries. Sociodemographic factors, especially sex and year of transplantation, were associated with transfusion in solid organ recipients, possibly as surrogates for other causal clinical factors.

## 1. Introduction

Solid organ transplantation is a lifesaving procedure for patients with end-stage organ dysfunction [1]. Over 140,000 solid organ transplantations were performed worldwide in 2018, a number that has continuously increased from 41,259 in 2000. Among types of solid organ transplantations, kidney and liver transplantations account for approximately 60% and 23% of total transplantations, respectively [2], and the prognosis of transplantation has improved with better short- and long-term graft survival [3,4].

Sensitization is one of the important and serious problems in organ transplant recipients, which leads to risks of hyperacute rejection. Highly sensitized patients are often required to wait longer for a compatible donor or to be desensitized [5]. Sensitization could be caused by factors including previous transplant, pregnancy, or blood transfusion. Antigen load, which is included in leukocyte, red blood cells (RBCs), platelets, and monocytes in blood units, is one possible cause of sensitization related to blood transfusion [5,6]. A recent study suggested that RBC transfusion negatively affected liver transplantation recipient survival [7]. Based on these results, it has been suggested that an active effort to minimize blood transfusion is required to prevent sensitization, despite the relatively low risk in naïve recipients [6]. However, during and early after transplantation, high amounts of blood loss often necessitate blood transfusion because of surgical procedures involving major vessels and impaired coagulation despite technique improvements in surgery and anesthesiology. 

In Korea, the number of organ transplantations has rapidly increased and systematic management of donor and recipient candidates has been established by the government, covering almost 100% of transplantation surgeries in Korea [8]. However, efforts to investigate trends in transfusion related to organ transplantation are limited. Therefore, this study analyzed the status and trends of transfusion and its associated factors among liver and kidney transplantation—the two main types of organ transplantation in Korea using a population-based nationwide database.

## 2. Materials and Methods

This study used data from the nationwide claims database of the National Health Insurance Service (NHIS) between 2008 and 2017. The NHIS is mandatory universal health insurance in Korea that covers more than 98% of the population. The Korean population pays insurance contributions and receives medical services from health care providers; the NHIS pays health care providers based on the records in the fee-for-service system. Information on insurance eligibility, such as demographic information, and insurance contributions of the insured and claims data from health care providers, including diagnoses, procedures, prescription records, and costs, are collected in the NHIS database. The details of the NHIS and its database have been described elsewhere [9]. The study design and protocol were approved by the Institutional Review Board of Hanyang University College of Medicine, Korea (Approval no: HYI-18-110, Approval date: 14 June 2018). All procedures were performed in accordance with the seventh version of the Declaration of Helsinki. As this study was an analysis of the NHIS database, informed consent was not obtained.

A total of 10,858 and 16,191 naïve liver and kidney transplantation recipients, respectively, from January 2008 to December 2017 were identified based on operation code among the health insurance claims (Q8040-Q8050 for liver and R3280 for kidney). Patients with multiple transplantation codes or transplantation-related codes for other sites were excluded. For the remaining patients, we identified the presence of transfusion of RBCs, fresh frozen plasma (FFP), platelets (PLTs), and cryoprecipitates (CRPs) based on procedure code; X2021, X2022, X2031, X2032, X2131, X2132, X2901, X2092, X2512, X2111, X2112, and X2515 for RBCs; X2041, X2042, X2051, and X2052 for FFP; X2081, X2082, X2121, X2122, X2501, X2516, X2511, X2513, and X2506 for PLTs; X2061 and X2062 for CRPs; and other codes between X001 and X3999 for others (whole blood, plasma, etc.). 

The presence of transfusion and the presence, number, and amount of each type of transfusion (WBCs, RBCs, FFP, PLTs, CRPs) were noted. We identified transfusions related to organ transplantation surgery by restricting the dates of the transfusion codes to those from 30 days before the date of operation until the end of hospitalization for the operation. Transfusion records more than 30 days before the date of operation or after the hospitalization period for the operation were excluded. The transfusion codes in each claim within the pre-defined period were noted. The amount of transfusion was the sum of the units of each type of transfusion, while the average amount of transfusion was the amount of transfusion divided by the number of recipients who received that blood component. 

The baseline characteristics of liver and kidney recipients by the presence of transfusion (transfusion rate) and chi-square *p*-values are presented. The characteristics considered were sex, age group, income, presence of disability, residential area, and year of transplantation. The presence of a disability was defined when people had a grade ‘yes’ for disability in the NHIS database, which is an evaluation system used to establish the level of governmental support for disabled people. For liver recipients, the type of donor (living or deceased) was additionally presented. The presence of transfusion and the presence, number, and amount of each type of transfusion were presented by year and the type of donor (living or deceased). Due to the lack of information on operation code in kidney recipients, the type of donor could not be determined. The odds ratios (ORs) and 95% confidence intervals (CIs) were determined to assess independent significant differences in the presence of transfusion and each type of transfusion with respect to the sex, age group, income, presence of disability, residential area, and year of transplantation (and type of donor in liver recipients). Statistical analyses were performed using SAS version 9.4 (SAS Institute Inc., Cary, NC, USA).

## 3. Results

### 3.1. Transfusion in Liver Recipients 

In this study, 96.4% of liver recipients received transfusions related to transplantation operation (Table 1). Among liver recipients, the proportions of patients receiving transfusions were significantly higher in women, younger patients (age <20 years), those with lower incomes, the more remote year of transplantation, and those receiving organs from deceased donors. No significant differences were observed in the proportions of patients receiving transfusion according to disability and residential area. 

**Figure 1:**
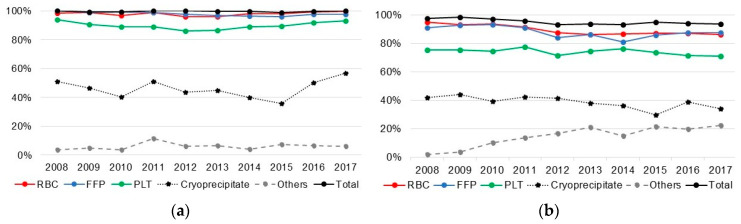

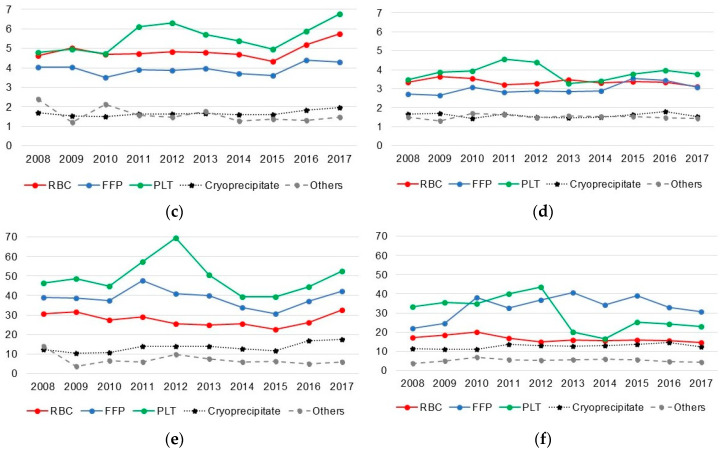
Proportion of transfusion, average count, and amount of transfusion in liver recipients by type of donor, 2008–2017. (**a**) Proportion transfusion among deceased liver donor recipients; (**b**) Proportion transfusion among living liver donor recipients; (**c**) Average count of transfusion among deceased liver donor recipients; (**d**) Average count of transfusion among living liver donor recipients; (**e**) Average amount of transfusion among deceased liver donor recipients; (**f**) Average amount of transfusion among living liver donor recipients. RBC: red blood cells; PLT: platelet; FFP: fresh frozen plasma.

The number and average number of transfusions and the amount and average amount of transfusion for each type of transfusion by year are shown in Figure 1. Among patients who received livers from deceased donors, 99.7% received blood transfusions. The most common blood component was FFP, followed by RBC and PLTs (Figure 1). In this study, 95.0% of liver recipients from living donors received transfusions. The most commonly transfused blood components were RBCs, FFP, and PLTs; however, the average amount of RBCs was lower. Although variable, the average amount of transfusion in deceased donor transplant recipients has been increased since 2015, but in living donor recipients, no trend was observed in average amount of transfusion.

Table 2 indicates the significant differences in transfusion needs and blood components by liver recipient characteristics after controlling for the effects of other factors. Among liver recipients, women showed increased odds for transfusion (OR = 3.81 (95% CI = 2.70–5.39)) of all types of transfusion (RBCs, FFP, PLTs, and CRP), especially RBCs. The increments in FFP, PLT, and CRP transfusion in women were less significant (OR < 2). Compared to liver recipients aged 50–59 years, those aged 20–39 years received transfusion less often (OR = 0.63 (95% CI = 0.41–0.97)) and received fewer PLT transfusions (OR = 0.66 (95% CI = 0.55–0.80)). Although there were no significant differences in the total number of transfusions in recipients aged <20 years, they received more RBCs (OR = 1.76 (95% CI = 1.03–3.01)) but fewer FFP, PLTs, and CRPs. Compared to recipients with incomes in the fourth quartile, those with lower-income quartiles showed higher proportions of patients receiving transfusion and each blood component transfused (OR range = 1.42–1.59). During the study period, the number of transfusions and transfusion of all types decreased by approximately 10% with year increment, based on the OR (Table 2) and the number of transfusions in each year (Table 1). Patients who received a liver from a deceased donor had much higher odds of overall transfusion (OR = 16.67, 95% CI = 8.33–33.33) and all types of transfusion (OR range = 1.39–6.25). Differences in transfusion rate by residential area were not consistent in most areas; however, recipients living in Jeju-do received significantly more transfusions (OR = 7.60 (95% CI = 1.04–55.42)) and blood components. 

### 3.2. Transfusion in Kidney Recipients

A total of 59.7% of kidney recipients received transfusions related to transplantation operation (Table 1). Higher transfusion rates were observed in kidney recipients who were female, the more remote year of transplantation, and older. In contrast to liver recipients, the transfusion rate did not differ by income group but did differ by residential area. 

**Figure 2:**
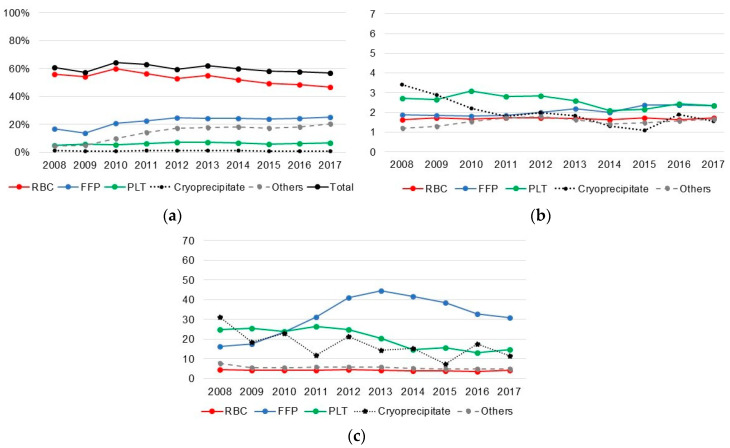
Proportion of transfusion, average count, and amount of transfusion in kidney recipient, 2008–2017. (**a**) Proportion transfusion among kidney recipients; (**b**) Average count of transfusion among kidney recipients; (**c**) Average amount of transfusion among kidney recipients. RBC: red blood cell; FFP: fresh frozen plasma; PLT: platelet.

Among kidney transplant recipients, RBCs were the most commonly transfused blood component; however, the average number and amount were lower than those for the other blood components (Figure 2). While the average amount of PLTs decreased, the other blood components did not show any trends. 

The differences in transfusion and blood components transfused according to the kidney recipient characteristics are shown in Table 3. Female kidney recipients received more transfusions (OR = 1.56 (95% CI = 1.47–1.67)) and all transfusion components (OR range = 1.37–1.67) than did male recipients. Among kidney recipients, as age decreased, the odds for transfusion also decreased. Income was not associated with transfusion needs; however, patients with income in the first to third quartiles received more FFP transfusions than those with income in the fourth quartile (OR = 1.3–1.4). Patients with income in the first quartile received fewer PLT transfusions (OR = 0.80 (95% CI = 0.65–0.99)). Despite the lack of differences in transfusion needs according to disability, patients without disability received fewer RBCs and PLTs and more FFP. The ORs of receiving transfusion and RBCs decreased during the study period (OR per year increment =0.98 (95% CI = 0.96–0.99) and 0.95 (95% CI = 0.94–0.96), respectively); however, the OR for FFP transfusion increased (OR = 1.05 (95% CI = 1.04–1.06)). Despite the inconsistent differences according to the residential area, kidney recipients living in Busan and Gyeongsangnam-do received more transfusions and approximately 1.5-fold higher amounts of most of the blood components.

## 4. Discussion

The results of this study showed the transfusion rates and transfusion amounts related to transplantation surgery among liver and kidney transplantation recipients in Korea from 2008 to 2017. Despite the decreasing trend of transfusion during the study period, most of the liver recipients and nearly all liver recipients who received organs from deceased donors, and approximately 60% of kidney recipients received blood transfusions. Studies have reported various transfusion rates in organ recipients. Studies with relatively lower transfusion rates have shown that approximately 75–80% of liver recipients did not receive any blood products and only 0.5 or fewer mean RBC units were required in 500 liver transplantations [10,11,12]. Another study reported that 30% of kidney recipients received transfusion [13,14]. Other studies showed higher proportions of intra-operative transfusions in which the average number of RBC and FFP units transfused intraoperatively were 8.5 and 10.3, respectively [15], while another study reported that more than 90% of liver recipients received transfusion [16]. A recent study also showed that only 1.7% of liver recipients did not receive transfusion [17]. Due to the high proportion of transfusions in liver recipients, previous studies on liver transplantation focused on not transfusion itself but rather on massive transfusion with various definitions [15,16,18]. Regarding kidney transplantation, some studies showed that approximately 50% of kidney recipients were transfused [19,20].

Due to the increased morbidity and mortality related to perioperative transfusion, studies have investigated the risk factors of transfusion and identified patients at high risk of requiring a transfusion, especially among liver recipients [2,12,15,16,18]. These studies were conducted in hospital settings and focused on clinical risk factors such as laboratory parameters or the severity of underlying diseases. In contrast, the present study analyzed a nationwide population, with a focus on the associations of sociodemographic factors with transfusion. The higher perioperative transfusion rate in women among both liver and kidney recipients may be caused by low hemoglobin and anticoagulant levels in women compared to those in men [21]. The recent decrease in the odds of transfusion during surgery in both liver and kidney recipients may reflect efforts to decrease transfusion during transplantation to reduce possible consequences of transfusion. Several studies reported an association between increasing age and massive transfusion; however, another study suggested that age was a surrogate factor for other unidentified risk factors based on the weak association [2,16]. In this study, weak and various associations between age and transfusion or type of transfusion in liver and kidney recipients were observed, supporting the previous suggestion that age was not a direct risk factor, but rather a surrogate for other risk factors [2,16]. 

Interestingly, liver recipients with low income were more likely to receive total blood, RBC, FFP, and PLT transfusions; however, among kidney recipients, income was not associated with transfusion except for FFP and PLT transfusion, although the directions of the association varied. Alcoholic liver disease was more common in lower-income patients and showed worse prognosis than other causes in liver recipients [22] and may explain the high transfusion rate in liver recipients with lower income. In kidney recipients, the distribution of the causes of chronic kidney failure varied by income level [23]; thus, the association between the causes and transfusion needs may not be uniform in these patients. 

The higher transfusion rate in liver recipients from deceased donors compared to that in liver recipients from living donors was consistent with previous findings [24]. In kidney transplantation, the transfusion rate was also higher in deceased donor transplants than in living donor transplants [20]. However, in the NHIS, the medical insurance code only identifies the donor type for liver transplantation. Thus, we could not determine whether the transfusion rate was higher in kidney recipients from deceased donors than that in kidney recipients from living donors. Previous studies also showed that transfusion during the transplantation surgery was associated with the outcome, complications, and mortality in both liver and kidney recipients [25,26]. In this study, the higher mortality rate in kidney recipients who received transfusion was consistent with the findings of a previous review (Table 4). However, among liver recipients, the higher mortality rate in transfused recipients was observed only among deceased donor transplant recipients; in living donor transplant recipients, the mortality was similar, irrespective of transfusion status (Table 4). This difference may be due to decreased adverse effects of transfusion owing to improved surgical and anesthesia techniques; however, the effect of donor type on survival remained. Otherwise, the high transfusion rate in liver recipients, even in living donor transplant recipients (approximately 95%), would not be enough to cause the observed differences in mortality. Among those receiving livers from deceased donors, only 10 did not receive transfusion and all were alive. Additional studies are warranted to further investigate the difference in mortality by transfusion in liver recipients and according to donor type in kidney recipients. 

Regarding residential area, an island geography and the lowest number of hospitals and hospital beds per person may explain the highest transfusion rate in liver transplantation in Jeju-do compared to other areas. For kidney transplantation, we found no reason for the higher transfusion rates observed in Busan and Gyeongsangnam-do, but a previous study on hip arthroplasty also showed a higher transfusion rate in these regions [27]. Therefore, further studies are required to reveal the reasons for the higher transfusion rates in relatively simple surgeries such as hip arthroplasty or kidney transplantation in these regions. 

To our knowledge, this study is the first to focus on sociodemographic factors in solid organ transplant recipients to assess transfusion needs and associated factors from a nationwide population-based database. By including all recipients from a population-based database, we could avoid possible selection bias. However, several limitations should be considered. First, we did not include recipients of multiple transplantations or re-transplantation, which may have resulted in an underestimation of the transfusion status or amount. Second, we did not consider recipients’ underlying diseases, cause of transplantation, or other known clinical factors related to transplantation. Thus, the associated sociodemographic factors in this study might not be independently associated with transfusion but may be surrogates for other causal factors. A recent study using the waiting list registration data from 11 liver transplantation centers in Korea reported that the most common cause of liver disease was hepatitis B, followed by alcoholic liver diseases and hepatitis C; in addition, approximately 50% of the patients had hepatocellular carcinoma with a mean end-stage liver disease score of 17.1 [28]. To capture all transfusions associated with transplantation surgery, we used transfusion codes registered between 30 days before the date of surgery and the discharge from hospital date. We applied various window periods and found that more than 95% of the transfusion codes were concentrated within 1 day before and after the operation date. A previous study also reported that the majority of transfusions were administered within the first month after the transplant [20]. Thus, the use of this period to define operation-related transfusion was relevant. Patients who received liver or kidney transplantation were identified by medical insurance codes in the NHIS database; thus, misclassification was possible. However, transplantations were reimbursed for by the NHIS and the number of liver and kidney transplantation per year was comparable to that reported in the national statistics based on the Korea national organ donation system. Thus, the definition of recipients based on medical insurance codes was valid. Due to the lack of information on the type of donor for kidney transplantation, it was impossible to identify the transfusion status according to the donor type (deceased or living), despite an increase in the rate of deceased kidney transplantation.

In this nationally representative study of transfusion needs among liver and kidney recipients, 96.4% of liver recipients and 59.7% of kidney recipients received blood transfusions, rates significantly higher than those reported previously despite variations between studies. The highest average transfused amounts were noted for PLT and FFP among both liver and kidney recipients. While the transfusion rates declined from 2008 to 2017 in both liver and kidney recipients, changes in transfusion amount were not observed. The transfusion rate in recipients who received organs from deceased donors was much higher than the rate in patients who received organs from living donors. The mortality rate in transfused patients was higher than that in non-transfused patients among deceased donor transplant recipients but did not differ in living donor transplant recipients. Despite the higher mortality rate in kidney recipients receiving transfusion compared to that in those without transfusion, further studies considering donor type (living or deceased) should be conducted in the Korean population. Moreover, considering the proportion of re-transplantation among the total liver or kidney transplantations in Korea (approximately 3% of the total liver transplantations and 8% of the total kidney transplantations), the status and effect of transfusion on the prognosis of patients who received liver or kidney re-transplantation needs to be assessed.

## Figures and Tables

**Table 1:** Transfusion needs by baseline characteristics of liver and kidney transplantation recipients in Korea, 2008–2017.

	Liver Transplantation (N = 10858)			Kidney Transplantation (N = 16191)		
	Not Transfused	Transfused	*p* ^a^	Not Transfused	Transfused	*p* ^a^
Sex						
Male	359 (4.7%)	7363 (95.4%)	<0.001	4282 (44.6%)	5325 (55.4%)	<0.001
Female	37 (1.2%)	3099 (98.8%)		2248 (34.1%)	4336 (65.9%)	
Age group (years)						
<20	7 (1.5%)	457 (98.5%)	0.026	183 (43.3%)	240 (56.7%)	0.001
20–39	27 (3.5%)	744 (96.5%)		1650 (42.3%)	2249 (57.7%)	
40–49	73 (3.1%)	2309 (96.9%)		1831 (40.9%)	2641 (59.1%)	
50–59	197 (4.1%)	4653 (95.9%)		2029 (39.3%)	3135 (60.7%)	
≥60	92 (3.9%)	2299 (96.2%)		837 (37.5%)	1396 (62.5%)	
Income (quartile)						
1st	54 (3.2%)	1614 (96.8%)	<0.001	1045 (41.0%)	1505 (59.0%)	0.183
2nd	54 (2.9%)	1826 (97.1%)		1013 (40.0%)	1521 (60.0%)	
3rd	75 (2.9%)	2491 (97.1%)		1447 (40.4%)	2131 (59.6%)	
4th	198 (5.0%)	3781 (95.0%)		2113 (41.1%)	3030 (58.9%)	
Missing	15 (2.0%)	750 (98.0%)		912 (38.2%)	1474 (61.8%)	
Disability						
No	364 (3.7%)	9418 (96.3%)	0.215	2439 (40.8%)	3547 (59.3%)	0.411
Yes	32 (3.0%)	1044 (97.0%)		4091 (40.1%)	6114 (59.9%)	
Residential area						
Seoul	82 (4.0%)	1992 (96.1%)	0.693	1317 (41.4%)	1867 (58.6%)	<0.001
Busan	24 (3.4%)	685 (96.6%)		417 (32.0%)	887 (68.0%)	
Incheon	13 (2.1%)	610 (97.9%)		337 (37.0%)	575 (63.1%)	
Daegu	19 (3.2%)	579 (96.8%)		347 (39.3%)	535 (60.7%)	
Gwangju	10 (3.2%)	307 (96.9%)		202 (45.6%)	241 (54.4%)	
Daejeon	10 (3.4%)	284 (96.6%)		186 (37.6%)	309 (62.4%)	
Ulsan	10 (3.7%)	259 (96.3%)		217 (51.4%)	205 (48.6%)	
Sejong	1 (6.7%)	14 (93.3%)		12 (52.2%)	11 (47.8%)	
Gyeonggi-do	93 (3.7%)	2453 (96.4%)		1598 (41.5%)	2257 (58.6%)	
Gangwon-do	12 (3.4%)	346 (96.7%)		177 (38.6%)	282 (61.4%)	
Chungcheongbuk-do	12 (3.7%)	309 (96.3%)		211 (46.6%)	242 (53.4%)	
Chungcheongnam-do	17 (4.1%)	399 (95.9%)		234 (40.1%)	350 (59.9%)	
Jeollabuk-do	14 (3.8%)	359 (96.3%)		233 (43.2%)	307 (56.9%)	
Jeollanam-do	21 (4.6%)	440 (95.4%)		226 (40.3%)	335 (59.7%)	
Gyeongsangbuk-do	27 (3.9%)	669 (96.1%)		327 (39.0%)	512 (61.0%)	
Gyeongsangnam-do	30 (4.6%)	619 (95.4%)		390 (38.4%)	625 (61.6%)	
Jeju-do	1 (0.7%)	138 (99.3%)		99 (45.0%)	121 (55.0%)	
Year of transplantation						
2008	15 (1.8%)	844 (98.3%)	<0.001	435 (39.3%)	673 (60.7%)	<0.001
2009	13 (1.5%)	883 (98.6%)		499 (42.1%)	687 (57.9%)	
2010	23 (2.4%)	935 (97.6%)		440 (35.9%)	787 (64.1%)	
2011	33 (3.1%)	1031 (96.9%)		584 (37.2%)	984 (62.8%)	
2012	54 (4.9%)	1047 (95.1%)		691 (40.6%)	1012 (59.4%)	
2013	47 (4.6%)	977 (95.4%)		636 (38.0%)	1038 (62.0%)	
2014	53 (4.8%)	1063 (95.3%)		700 (40.3%)	1039 (59.8%)	
2015	48 (3.9%)	1199 (96.2%)		759 (42.0%)	1048 (58.0%)	
2016	52 (4.0%)	1244 (96.0%)		897 (42.3%)	1224 (57.7%)	
2017	58 (4.5%)	1239 (95.5%)		889 (43.2%)	1169 (56.8%)	
Type of donor						
Deceased	10 (0.3%)	3110 (99.7%)	<0.001	-	-	
Living	386 (5.0%)	7352 (95.0%)		-	-	

**^a^** Chi-square *p*-value for the differences between patients who received transfusion and those who did not

**Table 2:** Factors associated with transfusion among liver recipients.

	Total	RBC	FFP	PLT	Cryoprecipitate
Sex					
Male	1 (ref)	1 (ref)	1 (ref)	1 (ref)	1 (ref)
Female	3.81 (2.70–5.39)	3.33 (2.67–4.15)	1.63 (1.38–1.92)	1.46 (1.30–1.63)	1.11 (1.02–1.21)
Age group (years)					
<20	1.38 (0.64–3.00)	1.76 (1.03–3.01)	0.45 (0.33–0.60)	0.15 (0.12–0.19)	0.78 (0.64–0.96)
20–39	0.63 (0.41–0.97)	1.05 (0.75–1.46)	1.02 (0.75–1.39)	0.66 (0.55–0.80)	1.32 (1.13–1.54)
40–49	1.06 (0.80–1.40)	1.08 (0.90–1.30)	1.13 (0.94–1.36)	1.08 (0.95–1.23)	1.16 (1.05–1.29)
50–59	1 (ref)	1 (ref)	1 (ref)	1 (ref)	1 (ref)
≥60	0.95 (0.73–1.23)	1.09 (0.91–1.31)	0.96 (0.81–1.14)	0.98 (0.86–1.11)	1.02 (0.92–1.13)
Income (quartile)					
1st	1.42 (1.04–1.94)	1.33 (1.07–1.65)	1.35 (1.10–1.66)	1.32 (1.13–1.53)	1.02 (0.91–1.15)
2nd	1.66 (1.21–2.27)	1.46 (1.18–1.81)	1.37 (1.13–1.67)	1.25 (1.09–1.44)	0.95 (0.85–1.07)
3rd	1.59 (1.21–2.10)	1.34 (1.11–1.62)	1.34 (1.13–1.60)	1.10 (0.97–1.24)	1.03 (0.93–1.14)
4th	1 (ref)	1 (ref)	1 (ref)	1 (ref)	1 (ref)
Disability					
No	1 (ref)	1 (ref)	1 (ref)	1 (ref)	1 (ref)
Yes	1.06 (0.73–1.56)	1.27 (0.97–1.67)	2.07 (0.84–1.39)	1.26 (1.06–1.52)	1.14 (1.00–1.30)
Residential area					
Seoul	1 (ref)	1 (ref)	1 (ref)	1 (ref)	1 (ref)
Busan	1.40 (0.88–2.25)	1.27 (0.92–1.75)	1.29 (0.95–1.77)	1.46 (1.17–1.82)	1.24 (1.04–1.48)
Incheon	2.43 (1.33–4.42)	0.96 (0.71–1.31)	1.90 (1.31–2.74)	1.06 (0.86–1.32)	0.53 (0.44–0.66)
Daegu	1.12 (0.67–1.88)	1.31 (0.90–1.90)	1.13 (0.80–1.59)	1.10 (0.88–1.39)	1.09 (0.90–1.31)
Gwangju	1.64 (0.83–3.22)	1.86 (1.13–3.05)	1.40 (0.91–2.14)	1.49 (1.10–2.01)	1.64 (1.29–2.08)
Daejeon	1.21 (0.61–2.38)	1.40 (0.85–2.31)	1.46 (0.90–2.34)	1.49 (1.08–2.05)	1.30 (1.01–1.66)
Ulsan	1.23 (0.62–2.43)	1.41 (0.85–2.33)	0.85 (0.57–1.29)	1.27 (0.92–1.75)	2.40 (1.85–3.12)
Sejong	0.88 (0.11–7.27)	0.80 (0.17–3.83)	2.11 (0.27–16.57)	1.07 (0.32–3.61)	2.04 (0.73–5.68)
Gyeonggi-do	1.17 (0.86–1.6)	1.15 (0.92–1.42)	0.97 (0.79–1.19)	1.40 (1.21–1.62)	0.98 (0.87–1.11)
Gangwon-do	1.35 (0.72–2.53)	1.10 (0.73–1.67)	0.91 (0.63–1.32)	1.13 (0.86–1.49)	1.16 (0.92–1.46)
Chungcheongbuk-do	1.28 (0.69–2.41)	1.12 (0.73–1.71)	1.44 (0.92–2.23)	1.21 (0.91–1.62)	1.23 (0.97–1.56)
Chungcheongnam-do	1.14 (0.67–1.97)	1.47 (0.97–2.23)	1.04 (0.73–1.49)	1.37 (1.05–1.78)	1.58 (1.28–1.96)
Jeollabuk-do	1.25 (0.69–2.24)	1.12 (0.75–1.67)	0.74 (0.53–1.04)	1.34 (1.01–1.77)	0.84 (0.67–1.07)
Jeollanam-do	1.13 (0.69–1.87)	1.38 (0.95–2.02)	1.24 (0.87–1.76)	1.30 (1.01–1.67)	1.64 (1.33–2.01)
Gyeongsangbuk-do	1.26 (0.80–1.98)	1.20 (0.88–1.65)	1.48 (1.07–2.04)	1.50 (1.20–1.87)	0.92 (0.77–1.10)
Gyeongsangnam-do	0.96 (0.62–1.48)	0.99 (0.72–1.35)	1.03 (0.76–1.39)	1.26 (1.01–1.57)	1.24 (1.03–1.48)
Jeju-do	7.60 (1.04–55.42)	2.25 (1.02–4.95)	2.84 (1.23–6.57)	1.80 (1.13–2.88)	1.89 (1.33–2.67)
Year of transplantation					
1–year increment	0.89 (0.85–0.92)	0.91 (0.88–0.93)	0.92 (0.89–0.94)	0.97 (0.95–0.99)	0.97 (0.96–0.99)
Donor type					
Deceased	16.67 (8.33–33.33)	6.25 (0.55–7.69)	6.25 (4.76–7.69)	3.44 (3.03–4.00)	1.39 (1.27–1.52)
Living	1 (ref)	1 (ref)	1 (ref)	1 (ref)	1 (ref)

RBC: red blood cell; FFP: fresh frozen plasma; PLT: platelet.

**Table 3:** Factors associated with transfusion among kidney recipients.

	Total	RBC	FFP	PLT	Cryoprecipitate
Sex					
Male	1 (ref)	1 (ref)	1 (ref)	1 (ref)	1 (ref)
Female	1.56 (1.47–1.67)	1.62 (1.52–1.73)	1.37 (1.27–1.48)	1.47 (1.29–1.67)	1.67 (1.23–2.28)
Age group (years)					
<20	0.83 (0.68–1.02)	0.97 (0.79–1.19)	0.63 (0.48–0.82)	0.60 (0.36–1.01)	0.62 (0.19–2.00)
20–39	0.84 (0.77–0.91)	0.83 (0.76–0.91)	0.82 (0.74–0.91)	0.83 (0.69–0.99)	0.56 (0.35–0.89)
40–49	0.90 (0.83–0.98)	0.92 (0.84–0.99)	0.95 (0.86–1.04)	0.93 (0.78–1.10)	0.90 (0.61–1.34)
50–59	1 (ref)	1 (ref)	1 (ref)	1 (ref)	1 (ref)
≥60	1.11 (1.00–1.23)	1.15 (1.04–1.27)	1.01 (0.89–1.13)	1.35 (1.12–1.62)	1.16 (0.73–1.84)
Income (quartile)					
1st	1.02 (0.92–1.13)	1.02 (0.92–1.12)	1.35 (1.10–1.66)	0.80 (0.65–0.99)	0.90 (0.52–1.54)
2nd	1.05 (0.95–1.16)	1.06 (0.96–1.17)	1.37 (1.13–1.67)	0.92 (0.75–1.12)	1.72 (1.11–2.67)
3rd	1.03 (0.94–1.12)	1.03 (0.95–1.13)	1.34 (1.13–1.60)	0.98 (0.82–1.16)	1.17 (0.75–1.80)
4th	1 (ref)	1 (ref)	1 (ref)	1 (ref)	1 (ref)
Disability					
No	1.00 (0.93–1.07)	0.87 (0.81–0.93)	1.42 (1.31–1.54)	0.82 (0.71–0.94)	1.27 (0.91–1.77)
Yes	1 (ref)	1 (ref)	1 (ref)	1 (ref)	1 (ref)
Residential area					
Seoul	1 (ref)	1 (ref)	1 (ref)	1 (ref)	1 (ref)
Busan	1.51 (1.31–1.73)	1.39 (1.21–1.58)	2.00 (1.74–2.31)	1.65 (1.31–2.09)	2.59 (1.55–4.33)
Incheon	1.23 (1.06–1.44)	1.46 (1.26–1.7)	0.66 (0.55–0.81)	0.99 (0.73–1.35)	1.24 (0.60–2.57)
Daegu	1.12 (0.96–1.31)	1.26 (1.08–1.47)	0.75 (0.62–0.91)	0.94 (0.68–1.3)	0.77 (0.32–1.87)
Gwangju	0.86 (0.70–1.05)	0.92 (0.75–1.12)	1.13 (0.89–1.43)	1.30 (0.89–1.91)	2.40 (1.12–5.13)
Daejeon	1.21 (1.00–1.48)	1.27 (1.05–1.54)	0.80 (0.62–1.02)	0.92 (0.61–1.39)	1.65 (0.72–3.79)
Ulsan	0.67 (0.55–0.83)	0.66 (0.53–0.81)	0.62 (0.47–0.82)	0.52 (0.3–0.91)	0.27 (0.04–1.96)
Sejong	0.75 (0.33–1.71)	0.64 (0.27–1.53)	0.32 (0.07–1.36)	–	–
Gyeonggi-do	1.01 (0.92–1.12)	1.05 (0.95–1.15)	0.95 (0.84–1.06)	0.96 (0.79–1.17)	0.86 (0.52–1.45)
Gangwon-do	1.16 (0.95–1.42)	1.17 (0.96–1.43)	1.26 (1.01–1.59)	0.83 (0.53–1.3)	0.74 (0.22–2.44)
Chungcheongbuk-do	0.83 (0.68–1.01)	0.87 (0.71–1.06)	1.07 (0.85–1.35)	0.73 (0.46–1.18)	1.23 (0.47–3.20)
Chungcheongnam-do	1.11 (0.92–1.32)	1.07 (0.89–1.27)	1.02 (0.82–1.26)	0.96 (0.66–1.41)	0.20 (0.03–1.46)
Jeollabuk-do	0.96 (0.80–1.16)	1.03 (0.85–1.24)	0.66 (0.51–0.85)	0.96 (0.65–1.42)	1.32 (0.54–3.21)
Jeollanam-do	1.10 (0.91–1.32)	1.06 (0.89–1.27)	1.08 (0.87–1.34)	0.78 (0.51–1.19)	0.64 (0.19–2.1)
Gyeongsangbuk-do	1.14 (0.98–1.34)	1.25 (1.07–1.46)	0.87 (0.72–1.05)	1.23 (0.91–1.66)	0.54 (0.19–1.55)
Gyeongsangnam-do	1.17 (1.01–1.35)	1.10 (0.95–1.27)	1.63 (1.39–1.90)	1.47 (1.13–1.91)	2.04 (1.12–3.69)
Jeju-do	0.89 (0.68–1.18)	1.12 (0.85–1.48)	0.67 (0.46–0.98)	0.52 (0.24–1.13)	0.53 (0.07–3.92)
Year of transplantation					
1–year increment	0.98 (0.96–0.99)	0.95 (0.94–0.96)	1.05 (1.04–1.06)	1.01 (0.99–1.03)	0.96 (0.91–1.02)

RBC: red blood cell; FFP: fresh frozen plasma; PLT: platelet.

**Table 4:** Associations between transfusion and mortality among liver and kidney recipients.

	Alive	Dead	*p*-Value
Liver recipients			
Not transfused	333 (84.1)	63 (15.9)	0.054
Transfused	8388 (80.2)	2074 (19.8)	
Deceased donor transplant recipients			
Not transfused	10 (100)	0 (0)	0.045
Transfused	2219 (71.4)	891 (28.7)	
Living donor transplant recipients			
Not transfused	323 (83.7)	63 (16.3)	0.905
Transfused	6169 (83.9)	1183 (16.1)	
Kidney recipients			
Not transfused	6319 (96.8)	211 (3.2)	<0.001
Transfused	9034 (93.5)	627 (6.5)	
